# Fulfillment of physical activity guidelines in the general population and frailty status in the elderly population: A correlation study of data from 11 European countries

**DOI:** 10.1007/s00508-018-1408-y

**Published:** 2018-11-12

**Authors:** Sandra Haider, Igor Grabovac, Thomas E. Dorner

**Affiliations:** Department of Social and Preventive Medicine, Centre for Public Health, Medical University of Vienna, Kinderspitalgasse 15/1, 1090 Vienna, Austria

**Keywords:** Demographic shift, Frailty, Aerobic physical activity recommendations, North-south gradient, Strength training

## Abstract

**Background:**

We report on the correlation between the proportion of people who fulfil the recommended amount of aerobic physical activity in the general population and the prevalence of frailty or prefrailty in the population ≥65 years in 11 European countries (Austria, Czech Republic, Denmark, Estonia, France, Germany, Italy, Luxembourg, Slovenia, Spain and Sweden). In a subgroup analysis, it was assessed if people who do aerobic physical activity also do strength training.

**Methods:**

Aggregated physical activity data were taken from the European Health Interview Survey with the minimum effective sample size of 90,036 participants. Data on frailty status were taken from the Survey of Health Ageing and Retirement in Europe (SHARE) study (*N* = 24,590). For the subgroup analysis, data of the Austrian Health Interview Survey (ATHIS) (*N* = 15,770) were included.

**Results:**

The results indicate a significant negative correlation between the proportion of people fulfilling the minimal aerobic physical activity recommendations (≥150 min/week) and the proportion of prefrail or frail people (R = −0.745; *p* = 0.008). The correlation between the optimal aerobic physical activity recommendations (≥300 min/week) and the proportion of prefrail or frail individuals was R = −0.691 (*p* = 0.019). In both data sets a north-south gradient was seen. Austrian data showed that 52.0% of the participants fulfilled the minimal aerobic physical activity recommendations and conducted strength training, whereas 18.4% did not fulfil the aerobic recommendations but performed strength training (*p* < 0.001).

**Conclusions:**

By taking into account that the number of people ≥65 years will increase in the future these results may be relevant in planning public health interventions for the whole population with the goal of reducing frailty in the elderly.

## Introduction

Frailty is a geriatric syndrome characterized by increased vulnerability and is associated with higher risk of disabilities, falls, morbidity, hospitalization, nursing home admission, and mortality [1–3]. As such it presents a substantial public health concern associated with increased healthcare costs [4, 5]. As regular physical activity has beneficial effects on maintaining functional status (e. g. muscle strength) and psychological issues (e. g. reduction of depression) as well as preventing chronic diseases (e. g. hypertension, cancer) and improving social outcomes (e. g. social network, social support), it is an important component in prevention and treatment of frailty [6, 7].

In adults, according to the national and international physical activity recommendations a multicomponent training regimen comprising aerobic and strength training is recommended [8, 9]. Concerning aerobic physical activity at least 150 min of moderate intensity or 75 min of vigorous intensity weekly is recommended. Optimally, 300 min of moderate intensity or 150 min of vigorous intensity physical activity are needed. This regular endurance training increases physical endurance, bone mineral density, fat mass and insulin sensitivity [10]. Furthermore, it decreases blood pressure, resting heart rate and basal insulin levels; however, endurance training does not significantly increase muscle mass [10]. Therefore, additional strength training twice a week is recommended [8, 9]. These recommendations also apply for the elderly population [8, 9]; however, in the aging population, strength training is particularly important, as sarcopenia, defined as low muscle mass in combination with low muscle strength or muscle performance [11], plays a relevant role. Both the decline in muscle mass [12, 13] and the decline in muscle strength [14–16] can be delayed with strength training [6, 17].

Based on this knowledge, it is postulated that the amount of physical activity in the general population is correlated with the prevalence of frailty in the elderly population. Thus, the aim of these analyses was to assess if countries where the adult population fulfils the recommended amount of aerobic physical activity is in correlation with the proportion of prefrail or frail elderly (≥65 years) individuals. Additionally, as especially strength training effects muscle mass and strength [17, 18] and reduces the risk of developing frailty, the study aimed to assess if people who reported doing aerobic physical activity also do strength training.

## Methods

### Data sources

For the purpose of this correlation study three data sources were used:

- European Health Interview Survey (EHIS): to assess aerobic physical activity, we used the aggregated data set of the EHIS (Wave 2; 2013), where data of subjects aged 15 years and older on time spent doing health enhancing aerobic physical activity were collected from 28 European countries [19]. The time spent in aerobic physical activity was assessed with the validated European Health Interview Survey Physical Activity Questionnaire (EHIS-PAQ) [20]. The percentages of persons reaching “0 min”, “1–149 min”, “≥150 min” and “≥300 min” of moderate aerobic physical activity were reported. In this data the amount of strength training was not assessed.
- Survey of Health, Ageing and Retirement in Europe (SHARE): for the assessment of the frailty status, the SHARE study (Wave 5; 2013) data were used [21]. In this study, data from persons aged ≥65 years and living in households were used. From all collected data, the items “exhaustion”, “weight loss”, “weakness”, “slowness” and “low activity” were used. Based on the SHARE frailty index [22], the discreet factor score was calculated and individuals were categorized as: 1) robust, and 2) prefrail or frail. Persons who did not answer or had missing values in any of the necessary items were excluded from the analysis.

Finally, data from 11 countries (Austria, Czech Republic, Denmark, Estonia, France, Germany, Italy, Luxembourg, Slovenia, Spain, Sweden) were included as both data on aerobic physical activity and the prefrailty/frailty prevalence were available. In these investigated 11 countries the minimum effective sample size of the EHIS was calculated to be 90,036 and data from 24,590 individuals of the SHARE study were available.

- Austrian Health Interview Survey (ATHIS): as strength training was not assessed in the EHIS, and the study aimed to investigate if people fulfilling the aerobic physical activity were also likely to fulfil the recommendation for strength training, a subsample analysis was carried out, including the individual-based data from the Austrian part of the EHIS, the ATHIS 2014, were used [23]. The dataset comprised 15,770 subjects aged 15 years or older. The proportion of individuals fulfilling the minimal aerobic physical activity recommendation and the proportion of people conducting strength training at least twice a week were used. Aerobic physical activity was also assessed with the validated European Health Interview Survey Physical Activity Questionnaire (EHIS-PAQ) [20], whereas strength training was assessed with the question “On how many days a week do you conduct exercises aiming to strengthen your muscles?”.

### Statistical analysis

The lowest and also the highest value of the EHIS data concerning the proportion of people in different aerobic physical activity categories are presented. Continuous normal distributed data of the SHARE study are presented as mean values and standard deviation (SD), and categorical variables in percentages, including minimum and maximum values seen in the respective countries. Spearman correlation coefficients were calculated to observe the associations between the proportion of individuals fulfilling the minimum recommendations for aerobic physical activity per week and the proportion of prefrail/frail individuals. The same was done with the criteria of optimal aerobic physical activity recommendation and the proportion of prefrail/frail individuals.

For the subgroup analysis of the ATHIS, cross-tabs were used and a χ^2^-tests were conducted to assess the association between the proportion of people fulfilling the 150 min of aerobic physical activity and the proportion of those doing strength training at least twice a week. For all calculations, a statistical probability of *p* < 0.05 was considered significant. Statistical software package SPSS 20.0 (SPSS Inc., Chicago, IL, USA) was used.

## Results

The EHIS data, providing the aerobic physical activity level of the people in the included 11 European countries, are shown in Table 1. Accordingly, the lowest proportion of all individuals who performed no aerobic physical activity at all was seen in Denmark and the highest in Italy. The proportion of people who performed 1–149 min of aerobic physical activity per week was reported as lowest in Spain and highest in Denmark. Additionally, the proportion of people performing ≥150 min of aerobic physical activity and who therefore fulfilled the minimum recommended aerobic physical activity was lowest in Italy and highest in Denmark. The country with the lowest proportion of people who performed ≥300 min per week aerobic physical activity was Italy, while the country with the highest being Denmark. Looking at the results of Austria, half of the participant performed ≥150 min/week of moderate aerobic physical activity (3rd place in the rankings) and about 30% stated to do ≥300 min/week (3rd place in the rankings).

**Table 1:** Aerobic physical activity level of 11 European countries

	0 min/week (%)	1–149 min/week (%)	≥150 min/week (%)	≥300 min/week (%)
Austria	25.1	24.6	50.4	29.4
Czech Republic	47.4	24.2	28.4	14.1
Denmark	18.7	**26.7**	**54.6**	**31.0**
Estonia	52.3	24.5	23.2	12.1
France	49.0	26.0	25.0	11.7
Germany	28.8	22.9	48.3	26.4
Italy	**65.0**	16.8	18.2	8.9
Luxembourg	36.5	21.9	41.6	21.9
Slovenia	39.1	23.0	37.9	22.4
Spain	51.0	15.0	34.0	21.1
Sweden	24.6	21.3	54.1	30.7

Data are taken from the European Health Interview Survey (EHIS; Wave 2; 2013), including all subjects aged 15 years. The highest proportion is marked in **bold**

Data of the SHARE study showed that participants in these investigated 11 countries had a mean age of 73.8 years (± 6.7 years) and 45.9% were male. Mean handgrip strength was 30.8 (± 11.1) kg. Exhaustion was reported between 31.4% in Austria and 52.7% in Estonia, while weight loss was indicated among 6.6% participants in Germany to 10.6% in Italy. Sweden had the lowest reported proportion of slowness with 9.6% while in Slovenia the reported levels were at 28.4%. Additionally, in Sweden 6.0% reported low activity level while the same was reported at 16.4% in Spain. The frailty prevalence of the included 11 European countries is shown in Table 2. Accordingly, the country with the highest frailty prevalence was Italy, the lowest in Sweden. The country with the highest prevalence of prefrailty was Spain, whereas Sweden once again had the lowest prevalence. Looking at the results, Austria had the 4th lowest prevalence of frailty and the 3rd lowest prevalence of prefrailty.

**Table 2:** Proportion in frailty categories in subjects ≥65 years in 11 European countries

Country	SHARE frailty index categories		
	Robust *n* (%)	Prefrail *n* (%)	Frail *n* (%)
Austria	1590 (74.9)	323 (15.2)	210 (9.9)
Czech Republic	1979 (69.5)	568 (20.0)	299 (10.5)
Denmark	1459 (77.8)	260 (13.9)	157 (8.4)
Estonia	1800 (63.0)	679 (23.8)	379 (13.3)
France	1495 (66.6)	466 (20.7)	285 (12.7)
Germany	1854 (75.5)	402 (16.4)	201 (8.2)
Italy	1349 (61.5)	469 (21.4)	377 (17.2)
Luxembourg	448 (71.1)	112 (17.8)	70 (11.1)
Slovenia	876 (63.7)	297 (21.6)	202 (14.7)
Spain	1892 (59.4)	**764 (24.0)**	**528 (16.6)**
Sweden	**2272 (81.3)**	379 (13.6)	142 (5.1)

Data are taken from the Survey of Health, Ageing and Retirement in Europe (SHARE) study (Wave 5; 2013). People were categorized as robust, prefrail or frail with the help of the SHARE frailty index [22]. The highest proportion is marked in **bold**

**Fig. 1:**
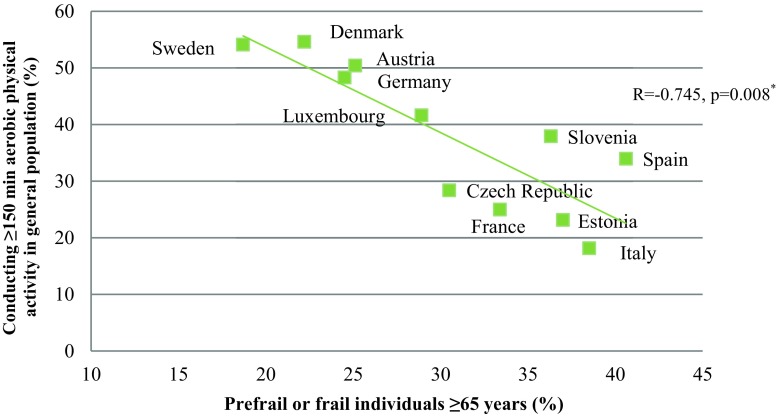
Correlation between the proportion of adult people conducting more than 150 min of aerobic physical activity/week and the proportion of prefrail or frail subjects ≥65 years in 11 European countries. (^*^Correlations were calculated using Spearman’s correlation coefficient)

**Fig. 2:**
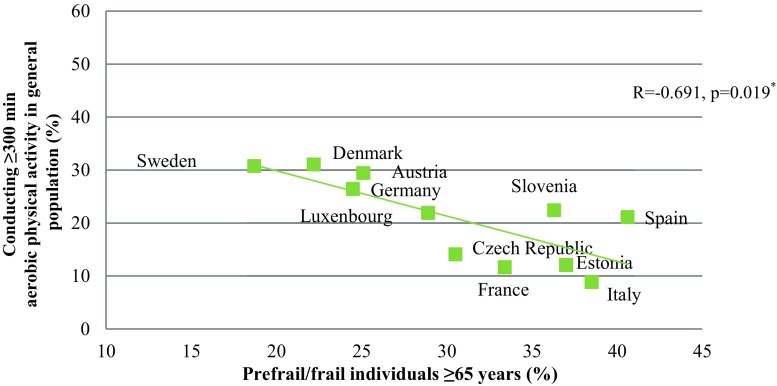
Correlation between the proportion of adult people conducting more than 300 min of aerobic physical activity/week and the proportion of prefrail or frail subjects ≥65 years in 11 European countries. (^*^Correlations were calculated using Spearman’s correlation coefficient)

Looking at the association between the ATHIS and the SHARE data, a significant negative correlation could be seen between the proportion of people fulfilling the minimal aerobic physical activity recommendations and the proportion of prefrail or frail people in the respective countries (R = −0.745; *p* = 0.008) (Fig. 1). There was also a significant correlation between the proportion of people fulfilling the optimal aerobic physical activity guidelines and the proportion of prefrail or frail subjects (R = −0.691; *p* = 0.019) (Fig. 2).

Concerning the association between aerobic physical activity and strength training, the ATHIS data showed that 52.0% of people fulfilling the minimum aerobic physical activity recommendations also conducted strength training at least twice a week, whereas 18.4% of the people who did not fulfil the minimum aerobic physical activity recommendations also performed strength training at least twice a week (*p* < 0.001).

## Discussion

The results showed that in European countries with a high proportion of physically active subjects in the general population, the proportion of prefrailty or frailty is low in the elderly population, and vice versa. This shows the importance of fulfilling at least the minimum physical activity recommendations in order to prevent frailty. These results are even more remarkable when taking into account that the examined populations were different, namely the entire adult population (≥15 years) with regard to the performed physical activity and on the other hand only the elderly (≥65 years) for the frailty status.

An issue that needs to be mentioned is that the correlation was found between aerobic physical activity and frailty status. As frailty status is largely associated with sarcopenia [24] that might especially be related to strength training and to a lower degree to aerobic physical activity, an even higher correlation was expected between strength training and frailty proportions; however, in a subgroup analysis only a weak association, although significant, was shown between achieving the recommended amount of aerobic physical activity and achieving the same for strength training (half of the subjects who fulfilled the aerobic guidelines did not meet the minimum for strength training). Nonetheless, further analyses should examine the association between strength training and frailty status across Europe.

The European north-south gradient of physically active subjects is in line with other studies [25]; however, in comparison to other northern European countries Estonia showed a higher frailty prevalence, which might also be explained by differences in the socioeconomic status in previous decades. Interestingly, in northern European countries people also reported more opportunities for physical activity, frequent more often sports clubs and fitness centers, and are more self-reliant on themselves and not on the local community to provide physical training possibilities [25]. This north-south gradient was also reported in data analyzing the frailty status across Europe, stating that socioeconomic factors such as education level are responsible for the differences [26, 27]. Thus, the observed differences in exercise behaviour and also in frailty status might be explained by the socioeconomic reasons, as less physical activity and higher prevalence of frailty are reported in poorer countries [26]. Additionally, cultural reasons that influence the different attitudes towards exercise and frailty status might be a further reason.

The major strength of this analysis was that a correlation study was performed on a European level for the first time. As the major limitation it should be mentioned that the study was only able to analyze the association between aerobic physical activity and prefrailty or frailty; however, as sarcopenia is related to frailty especially strength training is necessary to prevent frailty. It has also been taken into account that data on aerobic physical activity are self-reported. This might lead to an overestimation of physical activity [28].

## Conclusion

Taken together, the findings strengthen the postulation that community-based approaches aimed at achieving physical activity recommendations and creating exercise friendly environments. In particular, the results should be taken into account in southern European countries, when planning public health interventions. This may be important in increasing physical activity levels, prevention of frailty and prevention of other adverse health outcomes.
